# Outcomes of bypass surgery in asymptomatic moyamoya angiopathy: A multicenter study with propensity-score weighting

**DOI:** 10.1093/esj/23969873251365504

**Published:** 2026-01-01

**Authors:** Basel Musmar, Hammam Abdalrazeq, Joanna M Roy, Nimer Adeeb, Elias Atallah, Kareem El Naamani, Ching-Jen Chen, Roland Jabre, Hassan Saad, Jonathan A Grossberg, Adam A Dmytriw, Aman B Patel, Mirhojjat Khorasanizadeh, Christopher S Ogilvy, Andre Monteiro, Adnan Siddiqui, Gustavo M Cortez, Ricardo A Hanel, Alejandro M Spiotta, Anthony J Piscopo, David M Hasan, Mohammad Ghorbani, Joshua Weinberg, Shahid M Nimjee, Mohamed M Salem, Jan-Karl Burkhardt, Akli Zetchi, Charles Matouk, Brian M Howard, Rosalind Lai, Rose Du, Rawad Abbas, Abdelaziz Amllay, Alfredo Munoz, Nabeel A Herial, Stavropoula I Tjoumakaris, Michael Reid Gooch, Christina Notarianni, Bharat Guthikonda, Robert H Rosenwasser, Pascal Jabbour

**Affiliations:** Department of Neurological Surgery, Thomas Jefferson University Hospital, Philadelphia, PA, USA; Department of Neurological Surgery, Thomas Jefferson University Hospital, Philadelphia, PA, USA; Department of Neurological Surgery, Thomas Jefferson University Hospital, Philadelphia, PA, USA; Department of Neurosurgery, Louisiana State University Health Science Center, Shreveport, LA, USA; Department of Neurological Surgery, Thomas Jefferson University Hospital, Philadelphia, PA, USA; Department of Neurosurgery, University of Arizona College of Medicine, Tucson, AZ, USA; Department of Neurosurgery, The University of Texas Health Science Center, Houston, TX, USA; Department of Neurological Surgery, Thomas Jefferson University Hospital, Philadelphia, PA, USA; Department of Neurosurgery, Emory University, Atlanta, GA, USA; Department of Neurosurgery, Emory University, Atlanta, GA, USA; Department of Medical Imaging, University of Toronto Faculty of Medicine, Toronto, ON, Canada; Neuroendovascular Program, Massachusetts General Hospital & Brigham and Women’s Hospital, Harvard Medical School, Boston, MA, USA; Neuroendovascular Program, Massachusetts General Hospital & Brigham and Women’s Hospital, Harvard Medical School, Boston, MA, USA; Department of Neurosurgery, Beth Israel Deaconess Medical Center and Harvard Medical School, Boston, MA, USA; Department of Neurosurgery, Beth Israel Deaconess Medical Center and Harvard Medical School, Boston, MA, USA; Department of Neurological Surgery, Cooper University Health Care, Cooper Medical School of Rowan University, Camden, NJ, USA; Department of Neurosurgery, University of New York at Buffalo, Buffalo, NY, Buffalo; Department of Neurosurgery, University of New York at Buffalo, Buffalo, NY, Buffalo; Lyerly Neurosurgery, Baptist Health System, Jacksonville, FL, USA; Lyerly Neurosurgery, Baptist Health System, Jacksonville, FL, USA; Department of Neurosurgery and Neuroendovascular Surgery, Medical University of South Carolina, Charleston, SC, USA; Department of Neurosurgery and Neuroendovascular Surgery, Medical University of South Carolina, Charleston, SC, USA; Department of Neurosurgery, University of Iowa Hospital and Clinics, Iowa City, IA, USA; Department of Neurosurgery, Duke University, Durham, NC, USA; Department of Neurosurgery, Firoozgar Hospital, Tehran, Iran; Department of Neurosurgery, The Ohio State University Wexner Medical Center, Columbus, OH, USA; Department of Neurosurgery, The Ohio State University Wexner Medical Center, Columbus, OH, USA; Good Samaritan Hospital Medical Center, Babylon, NY, USA; Department of Neurosurgery, Hospital of the University of Pennsylvania, Penn Medicine, Philadelphia, PA, USA; Department of Neurosurgery, Hospital of the University of Pennsylvania, Penn Medicine, Philadelphia, PA, USA; Department of Neurosurgery, Yale University, New Haven, CT, USA; Department of Neurosurgery and of Radiology and Biomedical Imaging, Yale University, New Haven, CT, USA; Department of Neurosurgery, Yale University, New Haven, CT, USA; Department of Neurosurgery and of Radiology and Biomedical Imaging, Yale University, New Haven, CT, USA; Department of Neurosurgery, Emory University, Atlanta, GA, USA; Department of Neurosurgery, University of New York at Buffalo, Buffalo, NY, Buffalo; Neuroendovascular Program, Massachusetts General Hospital & Brigham and Women’s Hospital, Harvard Medical School, Boston, MA, USA; Department of Neurological Surgery, Thomas Jefferson University Hospital, Philadelphia, PA, USA; Department of Neurosurgery, Yale University, New Haven, CT, USA; Department of Neurological Surgery, Thomas Jefferson University Hospital, Philadelphia, PA, USA; Department of Neurological Surgery, Thomas Jefferson University Hospital, Philadelphia, PA, USA; Department of Neurological Surgery, Thomas Jefferson University Hospital, Philadelphia, PA, USA; Department of Neurological Surgery, Thomas Jefferson University Hospital, Philadelphia, PA, USA; Department of Neurosurgery, Louisiana State University Health Science Center, Shreveport, LA, USA; Department of Neurosurgery, Louisiana State University Health Science Center, Shreveport, LA, USA; Department of Neurological Surgery, Thomas Jefferson University Hospital, Philadelphia, PA, USA; Department of Neurological Surgery, Thomas Jefferson University Hospital, Philadelphia, PA, USA

**Keywords:** MMA, bypass, asymptomatic, stroke, IPTW%

## Abstract

**Introduction:**

Asymptomatic moyamoya angiopathy (MMA) is increasingly detected through noninvasive imaging; however, its optimal management remains controversial. This multicenter retrospective cohort study compared outcomes in asymptomatic versus symptomatic MMA patients undergoing surgical revascularization.

**Patients and methods:**

A total of 475 patients treated with bypass surgery across multiple academic centers were included, with 56 (11.8%) classified as asymptomatic and 419 (88.2%) as symptomatic. Baseline demographics, surgical characteristics, and outcomes-including perioperative stroke, intraoperative complications, and follow-up stroke events-were collected. Asymptomatic MMA was defined as the absence of any prior ischemic or hemorrhagic stroke, seizures, or other neurological symptoms at the time of diagnosis. Both unadjusted analyses and propensity score weighting using inverse probability of treatment weighting (IPTW) were performed to adjust for potential confounders.

**Results:**

In the unadjusted analysis, asymptomatic patients had significantly lower rates of all perioperative strokes (1.7% vs 11.4%; *p* = 0.05) and intraoperative complications (1.7% vs 11.2%; *p* = 0.05) compared to symptomatic patients. Additionally, follow-up stroke rates were lower in the asymptomatic group (1.7% vs 11.2%; *p* = 0.05). After IPTW adjustment, the reduction in intraoperative complications (OR: 0.08, 95% CI: 0.01–0.64; *p* = 0.01) and follow-up stroke rates (OR: 0.12, 95% CI: 0.01–0.91; *p* = 0.04) persisted, while differences in overall perioperative stroke were not statistically significant.

**Conclusion:**

Bypass surgery in selected asymptomatic MMA patients is associated with reduced intraoperative complications, and fewer follow-up stroke rates. These findings support the careful consideration of surgical intervention in asymptomatic patients, emphasizing the importance of patient selection for optimal outcomes.

## Graphical Abstract

**Graphical abstract:**
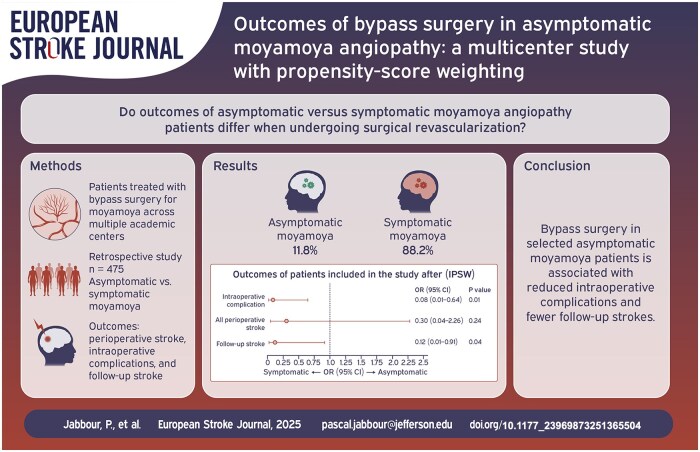

## Introduction

Moyamoya angiopathy (MMA) is a progressive cerebrovascular condition marked by gradual narrowing or occlusion of the terminal portions of the internal carotid arteries and their main branches.^1–7^ In response to this stenosis, an abnormal network of collateral vessels develops at the base of the brain—resembling a “puff of smoke” on angiographic imaging, a hallmark from which the disease derives its name.^8,9^ As these fragile moyamoya vessels compensate for impaired cerebral perfusion, they also pose a risk for rupture, making patients susceptible to both ischemic and hemorrhagic strokes.^8,9^ The clinical spectrum of MMA includes transient ischemic attacks (TIAs), cerebral infarction, and intracranial hemorrhage, with revascularization surgery commonly employed to reduce the risk of recurrent neurological events in symptomatic individuals.^10–17^

With the widespread use of noninvasive imaging such as magnetic resonance angiography (MRA), the identification of asymptomatic MMA has increased significantly.^8,18^ Recent reports show that asymptomatic cases now make up nearly 18% of diagnosed MMA patients, up from just 1.5% in the 1990s.^19,20^ Despite the absence of symptoms, several studies have demonstrated that asymptomatic MMA is not necessarily benign. Historical cohort data have reported annual stroke risks as high as 15% in patients managed conservatively, including both ischemic and hemorrhagic events.^8,21^

Currently, there are no established guidelines for the treatment of asymptomatic MMA. Some studies have suggested potential benefits of surgical intervention, including reduced mortality, while others have raised concerns about postoperative complications.^21–23^ Therefore, in this study, we aim to compare outcomes of asymptomatic and symptomatic MMA patients who underwent bypass surgery. The current study is the first and largest study to date to look at this issue as we did (asymptomatic vs symptomatic).

## Methods

### Study design and patient population

We conducted a multicenter, retrospective cohort study in accordance with the Strengthening the Reporting of Observational Studies in Epidemiology (STROBE) guidelines.^24^ Institutional Review Board (IRB) approval was obtained at each participating center. As the study used de-identified, retrospective data, the requirement for informed consent was waived. The underlying data for the study can be accessed through the corresponding author upon reasonable request.

This study included patients diagnosed with asymptomatic moyamoya angiopathy (MMA) who underwent surgical revascularization at 13 academic institutions across North America. Patients were enrolled retrospectively from January 2008 to December 2022. Asymptomatic MMA was defined as the absence of any prior ischemic or hemorrhagic stroke, seizures, or other neurological symptoms at the time of diagnosis. Diagnosis was confirmed using digital subtraction angiography (DSA).

Surgical indications for asymptomatic patients were based on a combination of imaging and clinical risk factors despite the absence of neurological symptoms. Specific inclusion criteria included: impaired cerebral perfusion demonstrated on SPECT, arterial spin labeling (ASL), or CT perfusion imaging; high-grade steno-occlusive lesions with poor collateral circulation on DSA; presence of silent infarcts or cerebral microbleeds on MRI; progressive angiographic worsening over serial imaging; and advanced Suzuki stage. All procedures were technically successful, confirmed via intraoperative assessment and postoperative imaging. Surgical technique (direct, indirect, or combined bypass) was selected at the discretion of the treating surgeon and institution. The choice of revascularization modality was influenced by several factors, including surgeon preference, institutional protocols, anatomical suitability, disease extent, and technical feasibility. Data were collected and analyzed per hemisphere. Decision-making was performed jointly between the treating neurosurgeons and patients following counseling on the risks and benefits of surgical versus conservative management.

### Data collection and definitions

A standardized data collection protocol was implemented across centers. Variables included patient demographics (age, sex, race), and comorbidities (hypertension, diabetes mellitus, smoking status, sickle cell disease). Radiographic information included laterality, vascular territory affected, and Suzuki grade. Operative variables included surgical side and type of bypass procedure. Perioperative stroke was defined as a new hypodensity on CT or a new lesion on diffusion-weighted MRI not present at baseline, accompanied by corresponding clinical symptoms. Intraoperative complications were also documented. Follow-up stroke was defined as the development of new neurological symptoms accompanied by corresponding radiographic evidence of acute infarction or hemorrhage on CT or MRI. Imaging surveillance during follow-up was performed in accordance with institutional protocols. All events were adjudicated by experienced neurologists and neurosurgeons at each site.

### Statistical analysis

All statistical analyses were performed using Stata version 17.0 (StataCorp, College Station, TX). Descriptive statistics were used to summarize baseline demographics, clinical characteristics, and outcomes across the asymptomatic and symptomatic moyamoya cohorts. Continuous variables were presented as medians with interquartile ranges (IQRs), and categorical variables as counts and percentages. Comparisons between groups were made using the Chi-square test for categorical variables, while Fisher’s exact test was applied when expected frequencies were fewer than five. For continuous variables, the Mann–Whitney U test was used due to non-normal distributions.

We used propensity score weighting to adjust for baseline differences between asymptomatic and symptomatic patients.^25^ Propensity scores were calculated using logistic regression, incorporating age, race, gender, hypertension, diabetes mellitus, smoking, surgery side, vascular territory, and procedure type.^26^ The PSWEIGHT package in Stata was used to generate these scores. Model fit was evaluated using the Hosmer–Lemeshow goodness-of-fit test.

To address the potential for bias from non-overlapping regions in the propensity score distribution, trimming was performed. Observations with propensity scores outside the range of 0.05–0.95 were excluded to ensure appropriate balance and reduce the influence of extreme values.^27^ Inverse probability of treatment weighting (IPTW) was then applied: patients in the asymptomatic group were weighted by the inverse of their propensity score (1/PS), and those in the symptomatic group by the inverse of one minus their score [1/(1–PS)]. To assess balance after applying IPTW, we evaluated the standardized mean differences of covariates before and after weighting. An absolute standardized mean difference (ASMD) of less than 0.10 was considered indicative of sufficient balance between the two cohorts (Supplemental Table 1). The distribution of propensity scores before IPTW is presented in Supplemental Figure 1. The weight distribution is presented in Supplemental Figure 2, which shows that the majority of IPTW weights are concentrated near 1, with no extreme outliers or evidence of undue inflation, suggesting stable estimation.

We assessed differences in outcomes between groups before and after weighting using univariable binary logistic regression for categorical outcomes and linear regression for continuous outcomes. Effect sizes were reported as odds ratios (ORs) or beta coefficients, along with corresponding 95% confidence intervals (CIs). All statistical tests were two-sided, and a *p*-value ⩽ 0.05 was considered statistically significant. Missing data were not imputed.

## Results

### Baseline characteristics

A total of 475 patients treated with surgical revascularization for MMA were included, comprising 56 (11.8%) asymptomatic and 419 (88.2%) symptomatic patients. The overall median age was 42 years (IQR 32–51), with the asymptomatic group showing a slightly higher median age of 48.5 years (IQR 28.5–53) compared to 41 years (IQR 32–51) in the symptomatic group; however, this difference did not reach statistical significance (*p* = 0.42). Gender distribution was similar between groups, with 39.2% of asymptomatic patients being male versus 30.0% in the symptomatic cohort (*p* = 0.16). Racial distribution was also comparable (*p* = 0.29), with Caucasian patients comprising 60.7% of the asymptomatic group and 47.4% of the symptomatic group. Other baseline comorbidities-including hypertension (44.6% vs 51.0%, *p* = 0.36), diabetes mellitus (21.4% vs 26.0%, *p* = 0.45), smoking status (33.9% vs 35.8%, *p* = 0.78), family history of moyamoya (1.7% vs 2.1%, *p* = 1.000), and sickle cell disease (3.5% vs 5.9%, *p* = 0.75)-did not differ significantly between the groups.

Operative characteristics were similar as well. Although there was a trend toward a higher proportion of right-sided surgeries in the asymptomatic group (49.0% vs 39.6%) and a lower rate of bilateral procedures (9.0% vs 16.5%), these differences were not statistically significant (*p* = 0.24). Distribution of vascular territories involved (*p* = 0.56), and procedure types – including direct, indirect, and combined revascularization (*p* = 0.74) – were also comparable between asymptomatic and symptomatic patients (Table 1).

**Table 1.** Baseline characteristics of patients with asymptomatic and symptomatic MMA treated with surgical revascularization.

Characteristics	Total (*n* = 475 )	Asymptomatic (*n* = 56)	Symptomatic (*n* = 419)	*p* Value
Age (years), median (IQR)	42 (32–51)	48.5 (28.5–53)	41 (32–51)	0.42
Gender, *n* (%)				0.16
Male	148/475 (31.1)	22/56 (39.2)	126/419 (30.0)	
Female	327/475 (68.8)	34/56 (60.7)	293/419 (69.9)	
Race, *n* (%)				0.29
Caucasian	233/475 (49.0)	34/56 (60.7)	199/419 (47.4)	
African-American	139/475 (29.2)	12/56 (21.4)	127/419 (30.3)	
Asian	59/475 (12.4)	4/56 (7.1)	55/419 (13.1)	
Hispanic	33/475 (6.9)	4/56 (7.1)	29/419 (6.9)	
Other	11/475 (2.3)	2/56 (3.5)	9/419 (2.1)	
Hypertension, *n* (%)	239/475 (50.3)	25/56 (44.6)	214/419 (51.0)	0.36
Diabetes mellitus, *n* (%)	121/475 (25.4)	12/56 (21.4)	109/419 (26.0)	0.45
Smoker, *n* (%)	169/475 (35.5)	19/56 (33.9)	150/419 (35.8)	0.78
Family history of Moyamoya, *n* (%)	10/475 (2.1)	1/56 (1.7)	9/419 (2.1)	1.000
Underlying disease, *n* (%)				0.94
No underlying disease	436/475 (91.7)	54/56 (96.4)	382/419 (91.1)	
Down syndrome	6/475 (1.2)	0/56 (0)	6/419 (1.2)	
Sickle cell disease	27/475 (5.6)	2/56 (3.5)	25/419 (5.9)	
Neurofibromatosis	3/475 (0.6)	0/56 (0)	3/419 (0.7)	
Sickle cell trait	3/475 (0.6)	0/56 (0)	3/419 (0.7)	
Surgery side, *n* (%)				0.24
Right	190/466 (40.7)	27/55 (49.0)	163/411 (39.6)	
Left	203/466 (43.5)	23/55 (41.8)	180/411 (43.8)	
Bilateral	73/466 (15.6)	5/55 (9.0)	68/411 (16.5)	
Suzuki grade, *n* (%)				0.29
I	13/421 (3.0)	2/39 (5.1)	11/382 (2.8)	
II	49/421 (11.6)	5/39 (12.8)	44/38.2 (11.5)	
III	135/421 (32.0)	18/39 (46.1)	117/382 (30.6)	
IV	128/421 (30.4)	9/39 (23.0)	119/382 (31.1)	
V	69/421 (16.3)	4/39 (10.2)	65/382 (17.0)	
VI	27/421 (6.4)	1/39 (2.5)	26/382 (6.8)	
Vascular territory, *n* (%)				0.56
MCA	310/470 (65.9)	34/56 (60.7)	276/414 (66.6)	
ACA	6/470 (1.2)	1/56 (1.7)	5/414 (1.2)	
Posterior circulation	7/470 (1.4)	0/56 (0)	7/414 (1.6)	
ICA	81/470 (17.2)	10/56 (17.8)	71/414 (17.1)	
Multiple territory	66/470 (14.0)	11/56 (19.6)	55/414 (13.2)	
Procedure type, *n* (%)				0.74
Direct revascularization	183/466 (39.2)	19/55 (34.5)	164/411 (39.9)	
Indirect revascularization	230/466 (49.3)	29/55 (52.7)	201/411 (48.9)	
Combined	53/466 (11.3)	7/55 (12.7)	46/411 (11.1)	

IQR: inter quartile range; MCA: middle cerebral artery; ACA: anterior cerebral artery; ICA: internal carotid artery.

### Outcomes before propensity score weighting

**Figure 1.**
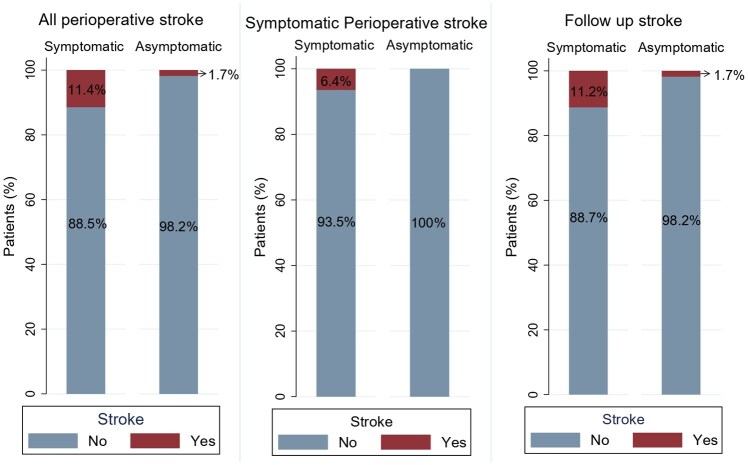
Unweighted (raw) comparison of stroke outcomes between symptomatic and asymptomatic moyamoya patients, illustrating rates of all perioperative stroke, symptomatic perioperative stroke, and follow-up stroke.

In the unadjusted analysis, the incidence of all perioperative strokes was significantly lower in the asymptomatic group, occurring in 1.7% (1/56) compared to 11.4% (48/419) in the symptomatic group (OR: 0.14, 95% CI: 0.01 to 1.03; *p* = 0.05; Figure 1). No symptomatic perioperative strokes or major symptomatic strokes were observed in the asymptomatic cohort (0%), whereas 6.4% (27/419) and 3.1% (13/419) of symptomatic patients experienced these events, respectively, although these differences did not reach statistical significance (*p* = 0.06 and *p* = 0.38).

**Figure 2.**
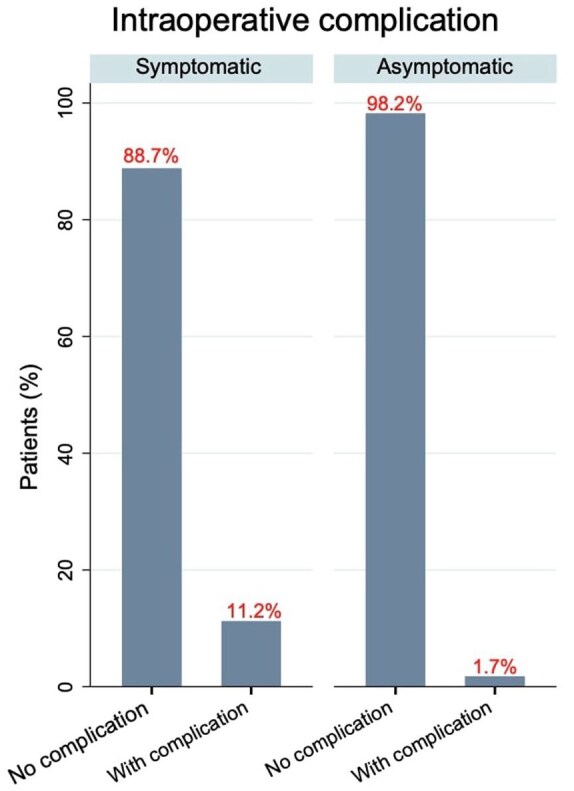
Unweighted (raw) comparison of intraoperative complication rates in symptomatic versus asymptomatic moyamoya patients.

Similarly, intraoperative complications were significantly fewer in asymptomatic patients (1.7% vs 11.2%; OR: 0.14, 95% CI: 0.01 to 1.06; *p* = 0.05; Figure 2). Mortality was low overall, with no deaths reported in the asymptomatic group compared to 0.9% (4/419) in symptomatic patients (*p* = 1.000). The median length of hospital stay was 3 days (IQR 2–5) for asymptomatic patients versus 4 days (IQR 3–5) for symptomatic patients (β: –0.82, 95% CI: –2.3 to 0.73; *p* = 0.29). During follow-up, strokes were documented in 1.7% of asymptomatic patients compared to 11.2% in symptomatic patients (OR: 0.14, 95% CI: 0.01 to 1.05; *p* = 0.05).

The median follow-up duration was comparable between groups at 15 months (IQR 8–39) for asymptomatic patients and 14 months (IQR 6–51) for symptomatic patients (β: –7.0, 95% CI: –22.58 to 8.40; *p* = 0.36; Table 2). Two symptomatic patients were lost to follow-up, while all asymptomatic patients completed follow-up.

**Table 2.** Outcome of patients with asymptomatic and symptomatic MMA treated with surgical revascularization.

Outcome	Asymptomatic (*n* = 56)	Symptomatic (*n* = 419)	Unadjusted model	
			OR/Beta (CI 95%)	*p* Value
All perioperative stroke, *n* (%)	1/56 (1.7)	48/419 (11.4)	0.14 (0.01 to 1.03)	0.05
Symptomatic perioperative stroke, *n* (%)	0/56 (0)	27/419 (6.4)	-	0.06^a^
Major symptomatic perioperative stroke, *n* (%)	0/56 (0)	13/419 (3.1)	-	0.38
Intraoperative complication, *n* (%)	1/56 (1.7)	47/419 (11.2)	0.14 (0.01 to 1.06)	0.05
Mortality	0/54 (0)	4/419 (0.9)	-	1.000^a^
Length of hospital stay (days), median (IQR)	3 (2–5)	4 (3–5)	−0.82 (−2.3 to 0.73)	0.29
Follow up stroke, *n* (%)	1/56 (1.7)	47/417 (11.2)	0.14 (0.01 to 1.05)	0.05
Type of follow up stroke, *n* (%)				1.000^a^
Ischemic stroke	1/1 (100)	39/47 (82.9)		
Hemorrhagic stroke	0/1 (0)	6/47 (12.7)		
Both	0/1 (0)	2/47 (4.2)		
Length of follow up (months), median (IQR)	15 (8–39)	14 (6–51)	−7.0 (−22.58 to 8.40)	0.36

IQR: inter quartile range.

^a^Fisher’s exact.

A descriptive subgroup analysis of outcomes by revascularization type across asymptomatic and symptomatic patients was performed (Supplemental Table 2). In the asymptomatic group, no symptomatic perioperative strokes, major strokes, or deaths occurred in any of the three surgical subgroups. One follow-up ischemic stroke occurred in the indirect bypass group. Among symptomatic patients, perioperative stroke rates were highest in the direct (12.8%) and indirect (11.4%) groups and lower in the combined bypass group (6.5%). Follow-up stroke was most frequent in the indirect bypass group (12.4%), followed by direct (11.0%), and was lowest after combined bypass (2.2%).

### Outcomes after inverse probability of treatment weighting (IPTW)

**Figure 3.**
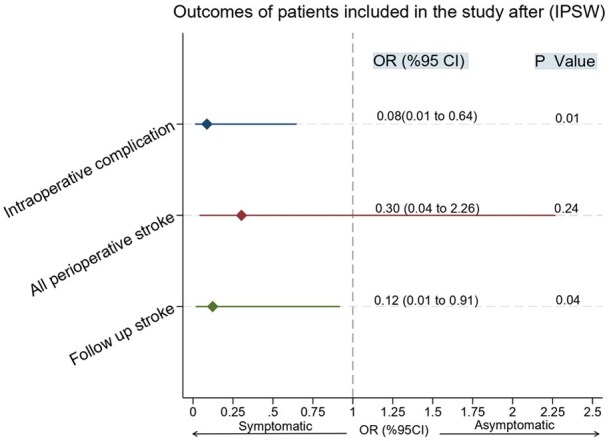
IPTW-adjusted outcomes comparing symptomatic and asymptomatic moyamoya patients, including adjusted odds ratios for perioperative stroke, intraoperative complications, and follow-up stroke.

After adjustment with IPTW, the difference in the risk of all perioperative strokes between asymptomatic and symptomatic patients was no longer statistically significant (OR: 0.30, 95% CI: 0.04 to 2.26; *p* = 0.24). However, the asymptomatic group continued to demonstrate a significantly reduced risk of intraoperative complications (OR: 0.08, 95% CI: 0.01–0.64; *p* = 0.01). The risk of follow-up stroke also remained lower among asymptomatic patients (OR: 0.12, 95% CI: 0.01–0.91; *p* = 0.04; Table 3; Figure 3).

**Table 3.** Outcome of patients with asymptomatic and symptomatic MMA treated with surgical revascularization adjusted model (after IPSW).

Outcome	(After IPSW) adjusted model	
	OR/Beta (CI 95%)	*p* Value
All perioperative stroke	0.30 (0.04 to 2.26)	0.24
Intraoperative complication	0.08 (0.01 to 0.64)	**0.01**
Length of hospital stay (days)	−1.21 (−2.31 to −0.12)	0.05
Follow up stroke	0.12 (0.01 to 0.91)	**0.04**
Length of follow up (months)	−2.60 (−14.81 to 9.60)	0.67

*P*-values in bold are statistically significant.

## Discussion

In this multicenter retrospective cohort study, we evaluated outcomes in asymptomatic versus symptomatic moyamoya patients who underwent surgical revascularization. Our findings indicate that asymptomatic patients exhibited significantly lower follow-up stroke rates and fewer intraoperative complications. Importantly, surgical bypass in asymptomatic patients was not associated with any symptomatic perioperative strokes, suggesting that, in selected cases, bypass surgery may reduce intraoperative complications and follow-up stroke rates without increasing the risk of symptomatic perioperative events.

The management of asymptomatic MMA remains a subject of considerable debate. Unlike symptomatic patients, deciding whether to perform surgery in the absence of overt clinical events poses a unique challenge.^8,21,28^ In a cohort of 113 adults with MMA, around 20% experienced disease progression over a 6-year period.^29^ Additionally, studies indicate that for adults managed conservatively, the annual risk of stroke ranges between 3.2% and 15.0%.^13,18,30,31^ These findings underscore the importance of carefully selecting patients for bypass surgery rather than waiting for them to become symptomatic. Our results contribute to this evidence base by indicating that bypass surgery in asymptomatic patients does not carry an increased risk of symptomatic perioperative strokes and may, in fact, lower the incidence of intraoperative complications and follow-up strokes.

In a study by Zeng et al., they compared asymptomatic patients treated conservatively with patients treated via bypass surgery.^28^ Three patients suffered from future clinical progression events in the conservative group while only one patient experienced transient ischemic attack in the surgical group. A study by Yamda et al. showed that none of the asymptomatic patients who underwent surgical treatment had any events during follow-up compared to six in the conservative end.^20^ Our study aligns with these findings, demonstrating that bypass surgery in selected asymptomatic patients is associated with lower odds of intraoperative complications and reduced follow-up stroke rates, without an increase in symptomatic perioperative strokes.

In another study by Lim et al.,^21^ bypass surgery in asymptomatic patients was linked to a reduced risk of death and ischemic stroke, although an increased risk of hemorrhagic stroke was observed. Our study partially aligns with these results, as none of the asymptomatic patients experienced hemorrhagic strokes during follow-up or symptomatic perioperative strokes.

Although our findings align with multiple Japanese and international studies, geographic practice patterns, patient selection criteria, and institutional variability may partially explain differences in reported outcomes across cohorts.^8,18,21^

The current study does not advocate for performing bypass surgery on all asymptomatic MMA patients but rather supports its use in selected individuals who may benefit from the procedure. Given the inherent risks associated with surgical interventions, our data suggest that it is reasonable for surgeons to consider bypass surgery in carefully selected asymptomatic patients instead of waiting for them to become symptomatic.

### Limitations

Our study has several limitations that must be considered. First, its retrospective design makes it inherently vulnerable to biases from data collection and patient selection, potentially affecting the generalizability of our findings. Despite our efforts to mitigate these biases with inverse probability of treatment weighting, unmeasured confounders may still be present. Second, because the study was conducted across multiple high-volume academic centers, there is notable variability in surgical techniques-particularly regarding the choice between direct and indirect bypass-as well as perioperative care protocols and follow-up practices. While prior meta-analyses have suggested that direct bypass may be preferred in adult moyamoya patients due to superior long-term outcomes,^32^ a recent large multicenter study by El Naamani et al. demonstrated comparable outcomes between the two approaches.^33^ The lack of standardized criteria for bypass selection across centers remains a limitation of our study. In addition, follow-up imaging protocols were not standardized across centers, which may have influenced stroke detection rates. While all events were adjudicated based on both clinical symptoms and radiographic confirmation, differences in surveillance frequency and modality may have introduced heterogeneity in stroke ascertainment. Moreover, although we performed a descriptive subgroup analysis stratified by revascularization type, the limited sample size of asymptomatic patients and the low frequency of outcome events within surgical subgroups precluded formal statistical interaction testing. The small number of asymptomatic patients limits the strength of conclusions that can be drawn about stroke prevention in this group. Third, our analysis was limited by the absence of detailed hemodynamic data, advanced imaging biomarkers, and genetic information (such as RNF213 mutation status), which could provide deeper insights into patient risk stratification. Although the study focused on surgically treated patients, we did not include a cohort of asymptomatic patients managed conservatively. This also limits our ability to directly compare outcomes between surgical and medical management strategies in this population. Finally, while our follow-up period was adequate to assess early postoperative outcomes, it may not fully capture the long-term progression and natural history of MMA.

## Conclusion

This study demonstrates that bypass surgery in selected asymptomatic moyamoya patients is associated with lower intraoperative complications and reduced follow-up stroke rates. These results support the careful consideration of surgical revascularization in asymptomatic patients, highlighting the importance of patient selection to optimize outcomes. Further prospective studies are needed to confirm these findings over the long term.

## Supplementary Material

supplementary_files_23969873251365504

## Data sharing statement

The relevant anonymized patient-level data are available on reasonable request from the authors.
