# Supplementary material for: Outcomes of bypass surgery in asymptomatic moyamoya angiopathy: A multicenter study with propensity-score weighting
Source: Eur Stroke J. 2026 Jan 1;11(1):23969873251365504. doi: 10.1093/esj/23969873251365504 (PMC12866215; doi:10.1093/esj/23969873251365504)
Supplement: supplementary_files_23969873251365504 [file supplementary_files_23969873251365504.zip › sj-docx-3-eso-10.1177_23969873251365504.docx]

**Supplementary Table 1: Standardized difference of co-variate included in IPSW in Asymptomatic and Symptomatic Moyamoya Angiopathy Treated with Surgical Revascularization.**

| **Variables** | **Mean, befor IPSW** | | **Standardized difference, before IPSW** | **Mean, Ater IPSW** | | **Standardized difference, After**  **IPSW** |
| --- | --- | --- | --- | --- | --- | --- |
|  | **Asymptomatic** | **Symptomatic** |  | **Asymptomatic** | **Symptomatic** |  |
| **Age** | 42.090 | 41.029 | 0.074 | 41.694 | 41.370 | 0.022 |
| **Gender** |  |  |  |  |  |  |
| ***Male*** | 0.381 | 0.289 | 0.201 | 0.277 | 0.314 | -0.079 |
| ***Female*** | 0.618 | 0.710 | -0.201 | 0.722 | 0.685 | 0.079 |
| **Race, n (%)** |  |  |  |  |  |  |
| ***Caucasian*** | 0.618 | 0.482 | 0.270 | 0.518 | 0.532 | -0.014 |
| ***African-American*** | 0.218 | 0.308 | -0.197 | 0.285 | 0.290 | -0.010 |
| ***Asian*** | 0.072 | 0.134 | -0.185 | 0.120 | 0.101 | 0.062 |
| ***Hispanic*** | 0.054 | 0.051 | 0.013 | 0.056 | 0.054 | 0.009 |
| ***Other*** | 0.036 | 0.022 | 0.093 | 0.018 | 0.020 | -0.014 |
| **Hypertension** | 0.454 | 0.519 | -0.130 | 0.469 | 0.494 | -0.049 |
| **Diabetes Mellitus** | 0.218 | 0.267 | -0.111 | 0.202 | 0.239 | -0.086 |
| **Smoker** | 0.327 | 0.362 | -0.073 | 0.344 | 0.348 | -0.008 |
| **Surgery side, n (%)** |  |  |  |  |  |  |
| ***Right*** | 0.490 | 0.397 | 0.190 | 0.437 | 0.438 | -0.002 |
| ***Left*** | 0.418 | 0.436 | -0.036 | 0.452 | 0.441 | 0.021 |
| ***Bilateral*** | 0.090 | 0.166 | -0.207 | 0.110 | 0.120 | -0.029 |
| **Vascular territory, n (%)** |  |  |  |  |  |  |
| ***MCA*** | 0.618 | 0.676 | -0.123 | 0.704 | 0.681 | .048 |
| ***ACA*** | 0.018 | 0.012 | 0.052 | 0.008 | 0.011 | -0.029 |
| ***Posterior circulation*** | 0 | 0.017 | -0.140 | - | - | - |
| ***ICA*** | 0.163 | 0.169 | -0.014 | 0.136 | 0.160 | -0.066 |
| ***Multiple territory*** | 0.20 | 0.125 | 0.219 | 0.151 | 0.146 | 0.013 |
| **Procedure type** |  |  |  |  |  |  |
| ***Direct Revascularization*** | 0.345 | 0.397 | -0.105 | 0.349 | 0.374 | -0.051 |
| ***Indirect Revascularization*** | 0.527 | 0.490 | 0.074 | 0.531 | 0.503 | 0.055 |
| ***Combined*** | 0.127 | 0.112 | 0.045 | 0.119 | 0.121 | -0.008 |

**Supplementary Table 2: Descriptive subgroup analysis of asymptomatic and symptomatic with outcome in each revascularization surgical type.**

| **Outcome** | **Direct** **Revascularization** (n=183) | | **Indirect Revascularization** (n=230) | | **Combined** **Revascularization** (n=53) | |
| --- | --- | --- | --- | --- | --- | --- |
|  | **Asymptomatic** | **Symptomatic** | **Asymptomatic** | **Symptomatic** | **Asymptomatic** | **Symptomatic** |
| **All perioperative stroke, n (%)** | 0/19 (0) | 21/164 (12.8) | 1/29 (3.4) | 23/201 (11.4) | 0/7 (0) | 3/46 (6.5) |
| **Symptomatic Perioperative stroke, n (%)** | 0/19 (0) | 14/164 (8.5) | 0/29 (0) | 10/201 (4.9) | 0/7 (0) | 3/46 (6.5) |
| **Major symptomatic perioperative stroke, n (%)** | 0/19 (0) | 6/164 (3.6) | 0/29 (0) | 5/201 (2.4) | 0/7 (0) | 2/46 (4.3) |
| **Intraoperative complication, n (%)** | 1/19 (5.2) | 14/164 (8.5) | 0/29 (0) | 25/201 (12.4) | 0/7 (0) | 8/46 (17.3) |
| **Mortality** | 0/19 (0) | 2/164 (1.2) | 0/29 (0) | 0/201 (0) | 0/7 (0) | 2/46 (4.3) |
| **Follow up stroke, n (%)** | 0/19 (0) | 18/163 (11.0) | 1/29 (3.4) | 25/201 (12.4) | 0/7 (0) | 1/45 (2.2) |
