# Supplementary figures and images for: Outcomes of bypass surgery in asymptomatic moyamoya angiopathy: A multicenter study with propensity-score weighting
Source: Eur Stroke J. 2026 Jan 1;11(1):23969873251365504. doi: 10.1093/esj/23969873251365504 (PMC12866215; doi:10.1093/esj/23969873251365504)

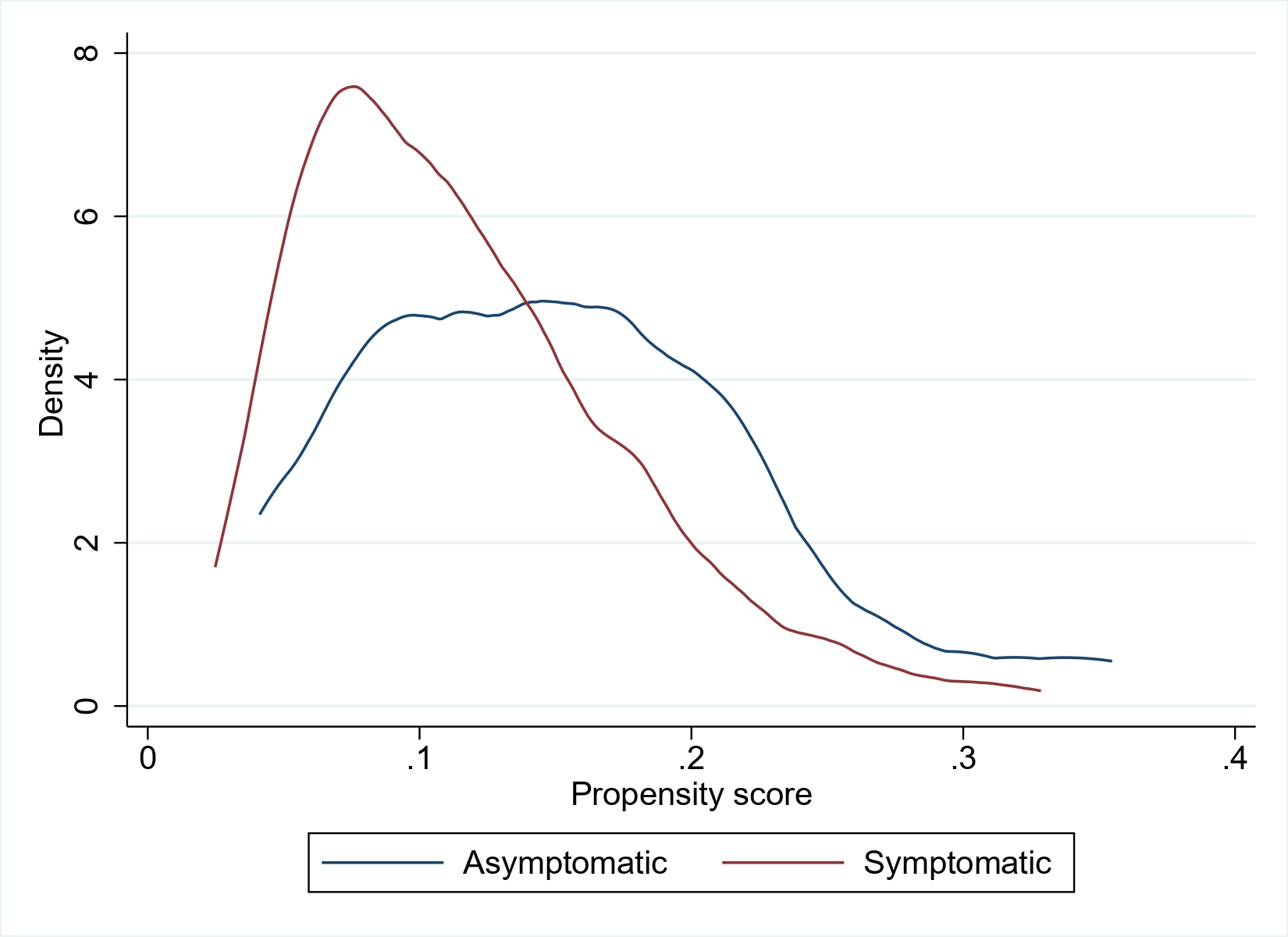

Supplement: supplementary_files_23969873251365504 [file supplementary_files_23969873251365504.zip › sj-jpg-1-eso-10.1177_23969873251365504.jpg]

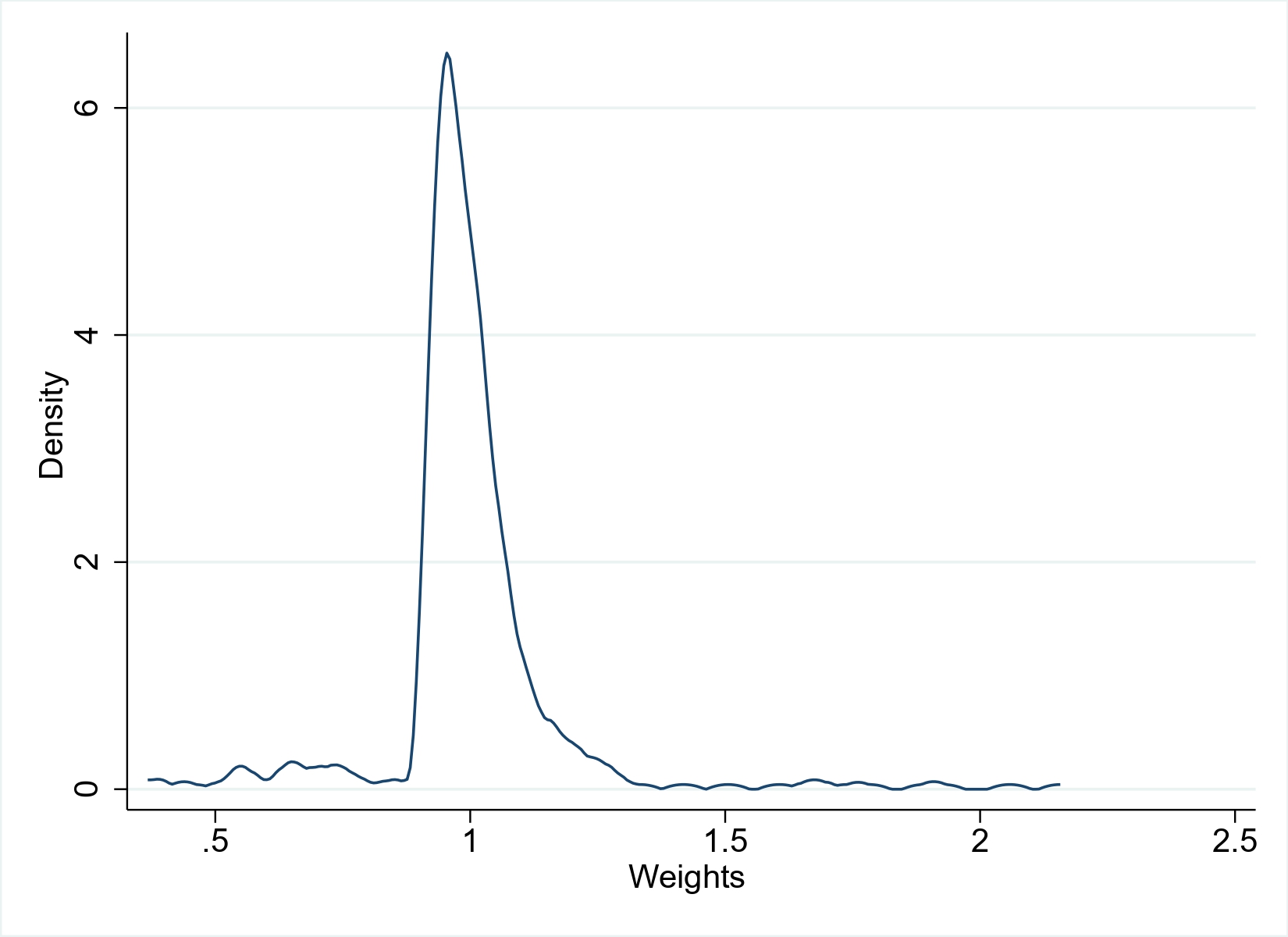

Supplement: supplementary_files_23969873251365504 [file supplementary_files_23969873251365504.zip › sj-jpg-2-eso-10.1177_23969873251365504.jpg]
